# Factor XI/XIa Inhibition: The Arsenal in Development for a New Therapeutic Target in Cardio- and Cerebrovascular Disease

**DOI:** 10.3390/jcdd9120437

**Published:** 2022-12-06

**Authors:** Juan J. Badimon, Gines Escolar, M. Urooj Zafar

**Affiliations:** 1 Cardiovascular Institute, Icahn School of Medicine at Mount Sinai, New York, NY 10029, USA; 2 Department of Hematopathology, Hospital Clinic, 08036 Barcelona, Spain

**Keywords:** factor XI, factor XI inhibitor, thrombosis, new drugs

## Abstract

Despite major advancements in the development of safer and more effective anticoagulant agents, bleeding complications remain a significant concern in the treatment of thromboembolic diseases. Improvements in our understanding of the coagulation pathways highlights the notion that the contact pathway—specifically factor XI (FXI)—has a greater role in the etiopathogenesis of thrombosis than in physiological hemostasis. As a result, a number of drugs targeting FXI are currently in different stages of testing and development. This article aims to review the different strategies directed towards FXI-inhibition with a brief summation of the agents in clinical development, and to comment on the therapeutic areas that could be explored for potential indications. Therapeutics targeting FXI/FXIa inhibition have the potential to usher in a new era of anticoagulation therapy.

## 1. Introduction

Thromboembolism and its associated complications remain a huge healthcare burden worldwide. The central role of thrombosis is observable in a variety of cardiovascular disorders, most notably in coronary artery disease (CAD), atrial fibrillation and stroke, peripheral arterial disease (PAD), and venous thromboembolism (VTE). Ischemic heart disease and stroke collectively are responsible for nearly 25% of all deaths worldwide [1], whereas estimates for the incidence rate for VTE, comprising deep vein thrombosis (DVT) and pulmonary embolism (PE), range from 115 to 269 per 100,000 people worldwide [2]. The impact of thrombosis extends beyond the cardiovascular arena and is increasingly being encountered in pathologies as diverse as cancer, immunological diseases, and even psychiatric disorders. Even among patients with human immunodeficiency virus (HIV) infection, who are living longer thanks to improvements in antiretroviral treatment, there is evidence of increased thrombosis [3], which contributes to their increasing morbidity and mortality from cardiovascular causes (6–15% of total mortality) [4,5].

The high human and financial cost of thromboembolic events underscore the need for newer and better therapeutic options for the management of thrombotic disorders. The challenge is in developing an agent that has potent antithrombotic effects but minimal bleeding risk, as it requires a very fine balancing act in modulating the hemostatic processes. Current anticoagulant options for clinical treatment of thrombotic disorders include antithrombin activators (unfractionated heparin), low molecular weight heparins (LMWHs and fondaparinux), vitamin K antagonists (VKA—warfarin), direct inhibitors of activated factor X (rivaroxaban, apixaban, edoxaban, betrixaban), and direct inhibitors of thrombin (hirudins, argatroban, and dabigatran). Although heparins and warfarin are low-cost options with a high degree of efficacy, both are associated with drawbacks that limit their clinical use. Heparin-induced thrombocytopenia, although infrequent, can be potentially lethal, and the development of osteoporosis and risk of contamination are additional factors to consider when using unfractionated heparin [6]. Some of these shortcomings have been reduced by LMWH and fondaparinux [6]. Warfarin presents the limitation of a narrow therapeutic window and major food- and drug-interactions [7]. The significant intra- and inter-patient variability of response of VKA makes frequent blood testing for dose-adjustment a cumbersome necessity. The last few years have provided a much-improved treatment option in direct oral anticoagulants (DOACs) that are convenient in administration while being potent and equally effective to VKA, often with a lower risk of bleeding [8,9,10,11]. Even so, the annual rate of major bleeding in patients on DOAC treatment remains significant [12], approximately 5% in elderly patients with atrial fibrillation (AF) [13]. This is partly why an unacceptably high proportion of AF patients—nearly one-third—do not receive the prophylactic anticoagulation they require. Even among those that do receive anticoagulation therapy, nearly half do not receive the proper doses [14]. The need for newer, safer anticoagulants is therefore high, and novel targets for therapeutic intervention are constantly under investigation.

## 2. Distinguishing Physiological Hemostasis from Pathological Thrombosis

Hemostasis is the normal, physiological process by which the clotting cascade seals up vascular damage to limit blood loss following injury. Thrombosis, on the other hand, encompasses various pathological conditions where the normally physiological clotting processes end up generating blood clot(s) inside the vascular lumen that are disruptive to the normal flow of blood. Thrombin generation and fibrin formation are the culminating steps in both hemostasis and thrombosis, but with important differences in the pathways involved.

Hemostasis is commonly triggered when tissue factor (TF) within the adventitial layer of blood vessels gets exposed to blood. Injury to vasculature that can lead to bleeding activates a series of soluble plasma proteins that act together in a cascade of enzyme activation events and culminate in the formation of platelet-fibrin clot(s). Because of the relatively high concentration of TF in such scenarios, the generation of thrombin is rapid and intense, quickly forming a hemostatic plug that seals the inciting TF away from blood. This disrupts the amplification of the coagulation processes through feedback mechanisms to the point of becoming pathological.

The concentration of TF in thrombosis is lower relative to hemostasis, but its duration of contact with blood components often lasts longer. Whether triggered by TF from disruption of an atherosclerotic plaque or activated monocytes/macrophages recruited to the site of injury or inflammation, or by implanted medical devices or neutrophil extracellular traps (NETs), these scenarios depend on the feedback mechanisms of the coagulation cascade for the growth and stabilization of the thrombus. This clot or thrombus can impede the flow of blood to the distal tissues and organs, leading to ischemia and necrosis, manifesting as clinical events including acute coronary syndrome, stroke, or deep vein thrombosis.

## 3. A Brief Review of the Classic Coagulation Cascade

**Figure 1:**
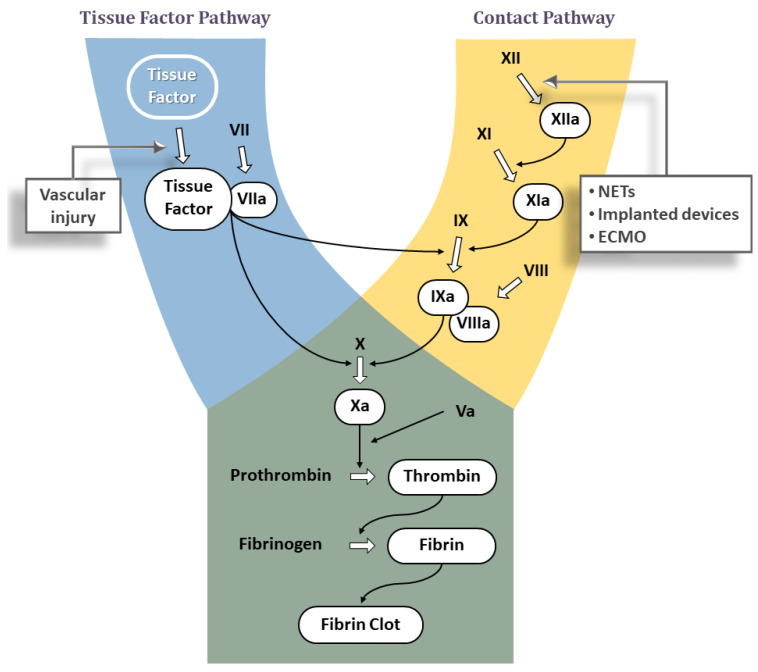
The classic model of the coagulation cascade with the Tissue Factor/extrinsic, the Contact/intrinsic and the common pathways. Triggering factors for both pathways are shown in square boxes. NETs: neutrophil extracellular traps, ECMO: extracorporeal membrane oxygenation.

The two major pathways for triggering blood clotting cascade are well known; (1) the tissue factor pathway and (2) the contact pathway. Both pathways trigger a series of cascading events that generate a blood clot (Figure 1) with the purpose to separate and seal the triggering agent from blood, thereby preventing its further contact with plasma components and arresting the thrombotic process.

### 3.1. Tissue Factor Pathway

This pathway is also known as the ‘Extrinsic’ pathway, as it is triggered by plasma components coming into contact with an agent that is extrinsic to blood (i.e., TF). The contact may happen when TF, normally embedded in the vascular wall, is exposed to blood due to rupture of a plaque, or when TF is expressed on the surface of cells active in inflammatory and immunological processes (e.g., monocytes and macrophages). 

Tissue Factor is an integral cell-membrane protein that forms a complex with the coagulation factor VIIa (FVIIa), normally present in plasma in the inactive zymogen form FVII. The TF:FVIIa complex is a potent activator of coagulation and converts factors IX (FIX) and X (FX) to the active forms FIXa and FXa, respectively (Figure 1). Each of these active enzymes assembles with its protein cofactor (FVIIIa and FVa, respectively) on suitable membrane surfaces to further propagate the coagulation cascade. The end result is a large burst of thrombin, the last serine protease in the clotting cascade. Thrombin not only converts fibrinogen into fibrin via limited proteolysis—which in turn assembles into a fibrin clot—but is also one of the most potent activators of platelets. The activation and aggregation of platelets contributes to the formation of a hemostatic plug. Additionally, thrombin also activates FV, FVIII, and FXI, the latter two of which are part of the contact pathway. Thus, the initial thrombin generated by the TF pathway can lead to activation of the contact pathway.

### 3.2. Contact Pathway

Also known as the ‘Intrinsic’ pathway, this pathway is triggered when blood comes into contact with anionic surfaces, such as extracellular DNA, RNA from activated or dying cells including neutrophil extracellular traps (NETs) released by activated neutrophils [15], or polyphosphates from the dense granules of activated platelets or microorganisms [16], or those on artificial surfaces [17]. This leads to a change in the conformation of plasma factor XII (FXII) into the active factor XII (FXIIa) [18,19]. FXIIa activates Prekallikrein to Kallikrein, which in turn reciprocally activates FXII to FXIIa in a positive feedback loop [20]. Downstream, FXIIa activates FXI to FXIa, which in turn leads to proteolysis of factor IX (FIX) to the active form (FIXa). The complex of FIXa and FVIIIa then activates FX to FXa at the point where the TF and contact pathways converge to form the final common pathway (Figure 1). The end result of all these interactions again is thrombin generation and formation of a blood clot.

The hemostatic process is kept in check by various inhibitory mechanisms that shut down the coagulation pathways, thereby localizing the hemostatic plug. These inhibitory mechanisms include proteins such as the tissue factor pathway inhibitor (TFPI) that inhibits FXa [21], activated protein C (APC), which degrades FVa and FVIIIa [22], and antithrombin (AT), which, in addition to factors IIa, Xa, and IXa [23], can also inhibit FVIIa and FXIa [24,25].

#### Significance of Contact Pathway in Thrombosis

**Figure 2:**
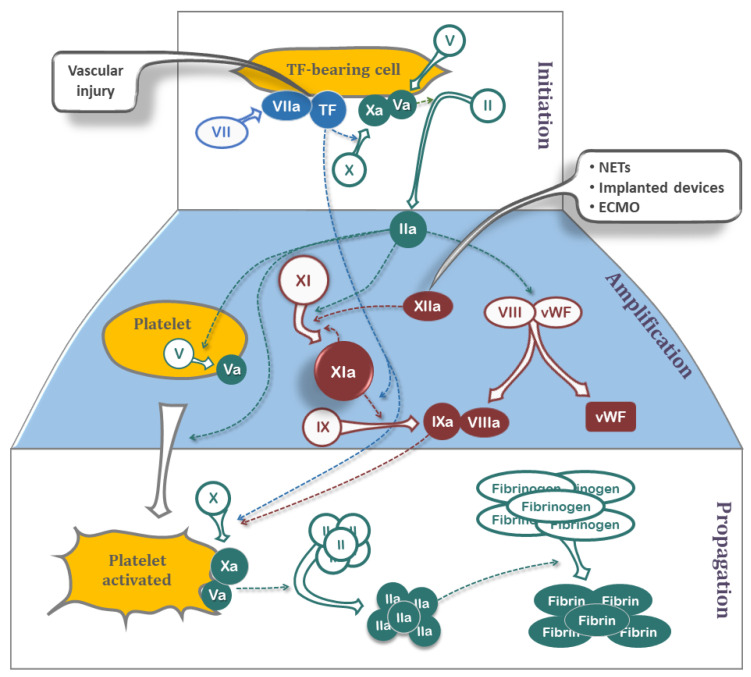
Cell-based coagulation model illustrating the Initiation, Amplification, and Propagation of the coagulation process. In the initiation phase, a small amount of thrombin and FIXa is generated on the surface of the tissue factor (TF)-bearing cell that then diffuse away towards platelets. In the amplification phase, this thrombin activates platelets (releasing factor Va from α granules), acts on vWF-VIII to release vWF and activated factor VIIIa, and generates activated factor XIa. The role of factor XI/XIa is primarily in the amplification stage where it activates factor IX and, in a feedback loop, promotes further activation of zymogen factor XI to active factor XIa. The propagation phase involves assembly of the various enzymes generated earlier to advance the process towards fibrin generation and clot formation. Components of the classic TF-, contact- and common-pathway are shown in blue, dark red, and green, respectively, along with triggers (black boxes) for each pathway.

Our understanding of the coagulation system in thrombotic pathophysiology has improved significantly in recent years, with the classical cascade being superseded by the cell-based model of coagulation (Figure 2). The TF pathway is understood to play a larger role in the ‘initiation’ and ‘propagation’ phases of coagulation, functioning more in normal hemostasis than in thrombosis. The contact pathway is more important in ‘amplifying’ the coagulation response, and despite its important role in clot formation in vitro, may contribute minimally to hemostasis in vivo, as supported by the lack of bleeding tendencies in patients deficient in FXII [26]. The contact pathway does appear, however, to have an important role in thrombotic disorders. Increased activity of plasma FXII, FXI, or kallikrein has been associated with atherosclerosis [27] and myocardial infarction [28,29], whereas severe FXI deficiency has been associated with reduced risk of stroke and deep vein thrombosis [30,31]. Deficiency in FXII in animal models has been reported to be protective against arterial thrombosis [32] and ischemic brain injury [33]. 

Selective modulation of the contact pathway theoretically should lower the risk of thrombosis without increasing bleeding. Development of drugs that act by inhibiting components of the contact pathway is currently in high gear, with factor XI (FXI), and to a lesser degree factor XII (FXII), being the most prominent targets [34,35,36]. Evidence from epidemiological studies supporting their role in thrombosis is stronger for FXI than it is for FXII [37].

## 4. Factor XI as a Therapeutic Target

Factor XI is a blood coagulation zymogen produced by the liver that is part of the early phase of the contact pathway [38]. It is converted to the active serine protease FXIa by thrombin, FXIIa, and by FXIa itself and in turn activates FIX to further advance the coagulation process [38]. FXI plays an important part in blood coagulation because its feedback activation amplifies in vivo thrombin generation and fibrin formation [39]. The additional thrombin formed via the FXI feedback loop also promotes the activation of Thrombin Activatable Fibrinolysis Inhibitor (TAFI), which increases the clot’s resistance to fibrinolysis, thereby helping to stabilize the formed clot.

The greater role of FXIa in thrombosis compared to hemostasis is evident from several epidemiological and genetic studies. Higher levels of circulating FXI levels are associated with increased risk for venous and arterial thrombosis, including stroke [40,41]. Deficiency of FXI (Hemophilia C, Plasma Thromboplastin Antecedent Deficiency, Rosenthal Syndrome) is rare and characterized by little to no bleeding tendency. Bleeding risk with factor XI deficiency selectively increases in tissues with high fibrinolytic activity (e.g., following dental surgery, tonsillectomy, and prostate surgery) [42]. Most frequent presentations involve nosebleeds or bleeding after tooth extractions. In fact, patients suffering from congenital FXI deficiency appear to have some degree of protection from thrombotic events, with lower rates of ischemic stroke and venous thromboembolism [30,43]. Moreover, hemorrhaging does not correlate with the levels of FXI in blood, i.e., bleeding is not restricted to patients with severe deficiency, and individuals with similar levels of FXI can experience different degrees of bleeding.

## 5. Pharmacologic Strategies for Factor XI Inhibition

**Figure 3:**
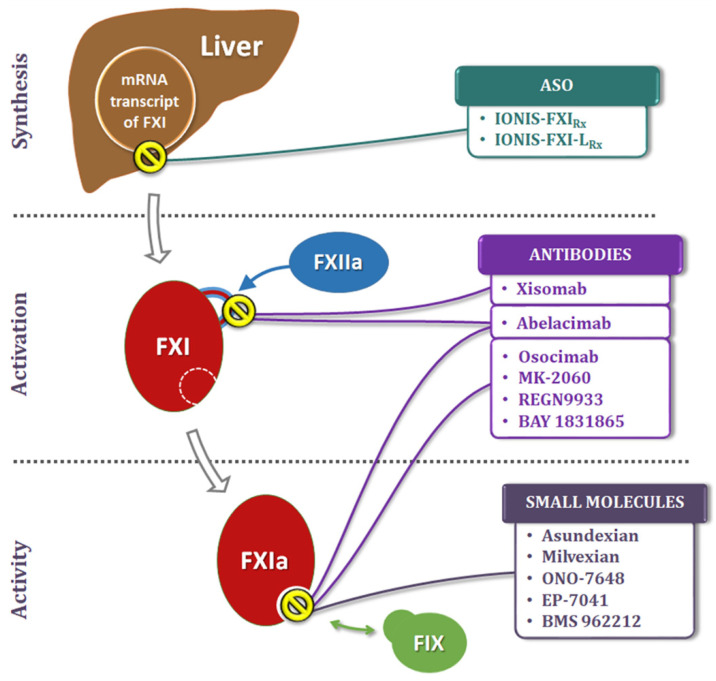
Sites of action (represented by the yellow circles) of the factor XI/XIa inhibitory drugs currently at different stages of clinical development. The anti-FXI ASOs (Antisense Oligonucleotides) block mRNA transcription of FXI in the hepatocytes, thus inhibiting its synthesis. Some monoclonal antibodies attach to the catalytic domain of FXI and block its FXIIa-mediated conversion to the active FXIa form, thus locking it in the inactive zymogen state. Most of the currently in development anti-FXIa antibodies act similar to the small molecule FXIa inhibitors and bind to the active site(s) on FXIa, thereby blocking its activity.

Given the larger role FXI is thought to play in thrombosis than in hemostasis, novel approaches to inhibit its generation and activity are being explored as new therapeutic strategies (Figure 3). These include: (a) Antisense Oligonucleotides (ASOs) that act on the liver to knockdown hepatic synthesis of FXI, (b) small molecules that target the FXI active site or the heparin allosteric site on FXIa, (c) monoclonal antibodies that act by blocking the activation or inhibiting the activity, and (d) Aptamers.

In addition to their varying mechanisms of action, these strategies also differ in their routes of administration (oral vs. parenteral), the onset of action, and the duration of effect. Parenteral administration is a requirement for ASOs, aptamers and monoclonal antibodies, whereas small molecule agents offer the option of either parenteral or oral administration. The varied onset and duration of action may present a broad set of treatment options depending on the pathology at hand; acute thrombotic events requiring quick-acting agents whereas longer-acting options, such as antibodies, would be more suitable for chronic prophylactic and preventative measures. Similarly, for conditions presenting a high risk of bleeding complications such as trauma or surgery, shorter-acting agents would be preferable. 

Inhibition of FXIa as a therapeutic option may also allow the possibility to easily reverse the effects of treatment, as has been tested successfully in animal models. In a rabbit AV-shunt model of thrombosis, the antithrombotic effects of a small molecule FXIa inhibitor (71.3 ± 5.2% lowering of thrombus weight vs. vehicle) were completely abolished by non-specific reversal agents (222% and 64% increase in thrombus weight vs. vehicle with FEIBA and NovoSeven, respectively) [44]. In another rabbit study, the addition of a specific reversal agent fully normalized the 210% prolongation in APTT produced by an anti-FXIa antibody [45]. This availability of reversing strategies for FXIa inhibition would be a significant advantage for this class of drugs, similar to the one available for some DOACs. Inability to reverse treatment effects can magnify the concerns about bleeding risks associated with any antithrombotic agent, thereby hampering its proper clinical utilization. As an example, although it is still possible to reverse the effects of antiplatelet drugs [46,47,48], the lack of a convenient and simple reversal strategy mandates that bleeding risk be always at the forefront of any discussion involving antiplatelet drugs.

A summary of FXIa inhibitors in more advanced stages of clinical development is presented in Table 1.

### 5.1. Antisense Oligonucleotides (ASOs)

Antisense Oligonucleotides are short, single-stranded nucleic acid sequences that pair with specific regions of mRNA and regulate its gene expression [49,50], thereby downgrading the hepatic synthesis of FXI. Their benefits include high specificity, predictable pharmacokinetics (PK), and long half-life. Furthermore, ASOs lack the drug–drug interactions commonly seen with conventional therapeutic agents. However, as nucleic acids in general are susceptible to degradation by nucleases, ASOs require some sort of chemical modification to confer nuclease resistance and enhance intracellular stability.

1. IONIS-FXIRx.

This agent (formerly known as ISIS 416858) is the ASO furthest in clinical development. Administered subcutaneously, IONIS-FXIRx has been shown to produce a concentration-dependent reduction in FXI antigen and activity levels [51]. In a phase II study of 315 patients undergoing total knee replacement, IONIS-FXIRx reduced the risk of postoperative VTE more than enoxaparin, without increasing the risk of bleeding. Rates of VTE were 27% and 4% among patients treated with 200 and 300 mg doses of IONIS-FXIRx, respectively, versus 30% in patients who received enoxaparin 40 mg once-daily [52]. Additionally, rates of major or clinically relevant non-major bleeding were also lower with IONIS-FXIRx (3% with both doses versus 8% with enoxaparin) [52].

IONIS-FXIRx has also been tested in 49 patients with end-stage renal disease (ESRD) requiring hemodialysis, where it produced a dose-dependent reduction in FXI antigen level and activity, and in aPTT without changing INR [53]. No drug-related serious adverse events or accumulation of the drug were observed after 12 weeks of treatment. A larger phase II study of the safety, PK, and pharmacodynamics (PD) was completed in 2019 in patients with ESRD, but no results are available to-date (NCT03358030).

2. IONIS-FXI-LRx.

A second-generation, ligand-conjugated antisense (LICA) agent named IONIS-FXI-LRx is also under clinical development. Its increased potency allows for once-monthly administration at lower doses, which helps to reduce the potential for injection-site reactions seen with IONIS-FXIRx. A phase II study of the safety, PK, and PD of IONIS-FXI-LRx in patients with ESRD was recently completed, but results are not yet available (NCT03582462).

**Table 1:** Inhibitors of FXI/FXIa in various stages of clinical development.

Compounds	Route	Stage	Indication	N	Status
ASO ^1^					
IONIS-FXI_Rx_	S.C. ^2^	Phase II	Total knee arthroplasty	315	Published [52]
		Phase II	ESRD ^4^	49	Published [53]
		Phase II	ESRD ^4^	213	Completed (NCT03358030)
IONIS-FXI-L_Rx_	S.C. ^2^	Phase II	ESRD ^4^	307	Completed (NCT04534114)
Small molecule					
Asundexian	Oral	Phase II	Myocardial infarction	1601	Published [54]
		Phase II	Ischemic stroke	1808	Published [55]
		Phase II	AF ^5^	753	Published [56]
		*Phase III*	*AF* ^5^*; Stroke and TIA* ^6^	*30,000*	*Announced [57]*
Milvexian	Oral	Phase II	Total knee arthroplasty	1242	Published [58]
		Phase II	Stroke and brain MRI ^7^		NCT03766581 [59]
ONO-7684	Oral	Phase I	PK ^8^ & PD ^9^ in healthy	48 + 24	Published [60]
EP-7041	I.V. ^3^	Phase II	Thrombocytopenia, COVID-19	90	Not recruiting (NCT05040776)
BMS-962212	I.V. ^3^	Phase I	PK ^8^ & PD ^9^ in healthy	691	Completed (NCT03197779)
Antibodies					
Abelacimab	S.C. ^2^	Phase II	Total knee arthroplasty	412	Published [61]
		Phase II	AF ^5^	1200	Not recruiting (NCT04755283)
		Phase III	Cancer-associated VTE ^10^	1655	Recruiting (NCT05171049)
		Phase III	GI/GU-associated VTE ^10^	1020	Recruiting (NCT05171075)
Osocimab	I.V. ^3^	Phase II	Total knee arthroplasty	813	Published [62]
Xisomab 3G3	I.V. ^3^	Phase II	ESRD ^4^	27	Published [63]
		Phase II	Thrombosis in chemotherapy	50	Recruiting (NCT04465760)
MK-2060	I.V. ^3^	Phase II	ESRD ^4^	489	Recruiting (NCT05027074)
REGN9933	I.V. ^3^	Phase I	PK ^8^ & PD ^9^ in healthy	72	Recruiting (NCT05102136)

^1^ Antisense Oligonucleotides; ^2^ subcutaneous; ^3^ intravenous; ^4^ end stage renal disease; ^5^ atrial fibrillation; ^6^ transient ischemic attack; ^7^ magnetic resonance imaging; ^8^ pharmacokinetic; ^9^ pharmacodynamic; ^10^ venous thrombo-embolism.

### 5.2. Small Molecules

#### 5.2.1. Small Molecules Targeting the Active Site on FXIa

A number of small molecules that inhibit FXIa activity by binding to the active site are in existence. These agents act by attaching to either S1, S2, or both pockets of FXIa [64].

1. Asundexian (BAY 2433334).

This small molecule is in the most advance stages of development among FXIa inhibitors, with results from three phase 2 studies published recently. The first to be published was a dose-finding trial that compared asundexian with placebo for the prevention of major adverse cardiac events in patients with recent acute MI on dual-antiplatelet therapy [54]. Patients (n = 1601) were randomized within 5 days of an MI to oral asundexian 10, 20, or 50 mg or placebo, given once-daily for 6–12 months in addition to aspirin plus a P2Y_12_ inhibitor. Over a year of follow-up, asundexian produced dose-dependent inhibition of FXIa activity without significant increase in bleeding (Bleeding Academic Research Consortium (BARC) bleeding type 2, 3, or 5: 7.6%, 8.1%, and 10.5%, respectively, with asundexian doses, vs. 9.0% with placebo) and had low rates of ischemic events (composite of cardiovascular death, MI, stroke, or stent thrombosis: 6.8%, 6.0%, and 5.5%, respectively, vs. 5.5% with placebo).

The second phase II trial, called PACIFIC-Stroke, compared asundexian 10, 20, or 50 mg vs. placebo for the secondary prevention of recurrent stroke. The study included 1808 patients with acute (<48 h) non-cardioembolic ischemic stroke, treated with single or dual antiplatelet therapy [55]. In this trial, asundexian did not reduce the primary efficacy endpoint—a composite of recurrent symptomatic ischemic stroke and MRI-detected covert brain infarcts at 26 weeks (19%, 22%, and 20% with asundexian 10, 20, and 50 mg, respectively, vs. 19% with placebo). Rates of major or clinically relevant non-major bleeding were 4%, 3%, and 4%, respectively, with asundexian vs. 2% with placebo.

The third phase II trial called the PACIFIC-AF trial, compared treatment with asundexian with apixaban in patients with nonvalvular atrial fibrillation (n = 753). Patients were randomly assigned to asundexian 20 or 50 mg once-daily or the standard 5 mg twice-daily dose of apixaban [56]. Both doses of asundexian had lower rates of major or clinically relevant non-major bleeding at 12 weeks vs. apixaban, with ratios of incidence proportions of the primary composite endpoint being 0.50 and 0.16 for asundexian 20 and 50 mg versus apixaban, respectively. Rates of thrombotic and cardiovascular events were reported to be comparable with the two treatments, but the thrombotic endpoints were exploratory due to sample size considerations.

The reporting of the asundexian phase II results has been followed by a recent announcement of a phase III development program. The OCEANIC program is expected to enroll up to 30,000 patients in two large multinational studies, OCEANIC-AF and OCEANIC-Stroke, involving atrial fibrillation and non-cardioembolic ischemic stroke or high-risk transient ischemic attack, respectively [57].

2. Milvexian (JNJ-70033093/BMS-986177).

This orally active agent is also in the advance stage of clinical development among FXIa inhibitors. In a phase II dose-finding trial in patients undergoing elective knee arthroplasty (AXIOMATIC-TKR; n = 1242), postoperative FXIa inhibition with milvexian was effective for the prevention of venous thromboembolism, with a dose-related response in both once-daily and twice-daily administrations [58]. With twice-daily administration of milvexian 25, 50, 100, and 200 mg, the dose-response relationship was statistically significant and the incidence of VTE significantly lower than the prespecified benchmark of 30% (21%, 11%, 9%, and 8%, respectively). In the same trial, rate of VTE with subcutaneous enoxaparin was 21%. Milvexian also showed promising results on the safety side, with low rates of any bleeding, major bleeding, or clinically relevant non-major bleeding relative to enoxaparin.

The findings from the second phase II trial (AXIOMATIC-SSP; n = 2366) for the prevention of new ischemic stroke in patients following acute ischemic stroke or transient ischemic attack were recently presented at the 2022 European Society of Cardiology Congress in Barcelona [59]. All patients received aspirin plus clopidogrel for 21 days, followed by aspirin alone thereafter. While the rate of the primary efficacy endpoint—a composite of ischemic overt stroke or covert stroke detected by brain MRI at 90 days—was numerically lower at the 50 mg and 100 mg twice-daily doses, there was no apparent dose-response. For clinical ischemic strokes (i.e., excluding covert brain infarction), milvexian doses from 25 to 100 mg twice-daily showed an ~30% relative risk reduction versus placebo. The rate of major bleeding was moderately increased with milvexian 50 mg twice-daily and above, but with no apparent dose-relation.

Based on the overall findings from the phase II studies, milvexian is moving towards further studies in phase III trials. Interestingly, investigation of antidotes to milvexian has also moved on to clinical stages (NCT04543383) after animal testing [44].

3. Other Small-Molecules in Early Development

A number of other oral and parenteral inhibitors of FXIa are in earlier phases of development. These include ONO-7684, an orally active agent that was well-tolerated in a phase I study with healthy volunteers. This study reported low overall incidence of adverse events with no evidence to suggest bleeding risk [60].

The parenteral small molecules under development include EP-7041. A placebo-controlled study to evaluate its safety, PK, and PD was conducted in healthy volunteers [65]. The drug was well-tolerated except for some cases of mild headache (23%) and infusion site bruising (7%). EP-7041 exhibited rapid onset–offset and dose-related increases of aPTT without affecting PT. Despite these positive results, there was no further development with this agent until 2021, when an IND application for its use as an investigational treatment for COVID-19 patients in ICU was accepted by the FDA (NCT05040776).

BMS-962212 is another parenterally administered, FXIa-inhibiting small molecule investigated in healthy participants. In testing of multiple doses, the drug was well-tolerated, with no bleeding events and mild adverse events in 17.6% participants [66]. Dose-dependent changes in aPTT and FXI were observed with maximal effects by approximately 2 h, and no changes in PT or INR.

#### 5.2.2. Small Molecules Targeting Heparin Allosteric Site on FXIa

This group of FXIa-directed agents exert their inhibitory effects by attaching to the heparin-binding site on the catalytic domain of FXIa. Given the structural similarities between the active sites of various serine proteases, it is believed that allosteric inhibition would have the advantage of being more specific. Some of the sulfated glycosaminoglycan (SPGG) mimetic compounds under development in this group not only exhibit a highly selective inhibition of FXIa than any other target in the coagulation cascade, but also display a reversal of their anticoagulant effects with FXI and serum albumin [67]. Protamine could also reverse the anticoagulant effects of SPGG, providing potential ways for the development of antidotes.

### 5.3. Monoclonal Antibodies

A number of monoclonal antibodies that block either FXI activation or FXIa protease activity are currently under development for the treatment of thrombotic disorders. The antithrombotic effects and bleeding risk of these antibodies are at various stages of testing.

1. Abelacimab (MAA868).

A monoclonal antibody that binds the procoagulant enzymatic site of both FXI (zymogen) and the active form FXIa [68]. By binding to the catalytic domain, abelacimab locks both the FXI and activated FXIa in an inactive, zymogen-like conformation, thereby taking them out of the coagulation system. In a phase I testing, the pharmacodynamic effects of a single subcutaneous administration lasted up to 4 weeks or longer, suggesting the possibility of a once-monthly dosing [68]. 

Abelacimab has been compared with enoxaparin for the prevention of VTE in patients undergoing elective knee arthroplasty (n = 412) in a phase 2 study with promising results. Patients were randomized to single intravenous administration of abelacimab 30, 75, or 150 mg, or to subcutaneous enoxaparin 40 mg [61]. Rates of VTE were 13%, 5%, and 4% with abelacimab doses, respectively, vs. 22% with enoxaparin, assessed by venography or objective confirmation of symptomatic events 8–12 days after the operation. Bleeding risk was low, with occurrence in 2% of cases with the lower two doses of abelacimab and none of the patients in the highest dose abelacimab or the enoxaparin groups. 

A larger phase II study to compare the bleeding risk of abelacimab vs. rivaroxaban in patients with AF at moderate-to-high risk of stroke (AZALEA-TIMI 71) is currently listed as ‘Active, not recruiting’ and plans to enroll 1200 patients (NCT04755283). Interestingly, the more advanced studies with this agent are in the prevention of cancer-associated VTE, with two active phase III trials comparing the efficacy of abelacimab vs. dalteparin (MAGNOLIA; NCT05171075) and vs. apixaban (ASTER; NCT05171049).

2. Osocimab (BAY 1213790).

This is a fully human IgG1 antibody. Its crystal structure analysis has shown a novel allosteric mechanism of action, with the antibody binding to a region adjacent to the FXIa active site, leading to structural rearrangements and blocking of activity. 

Osocimab has been compared with enoxaparin and apixaban for thromboprophylaxis in patients undergoing elective knee arthroplasty in the phase II FOXTROT trial (n = 813). A single intravenous administration of osocimab (given postoperatively at 0.3, 0.6, 1.2, or 1.8 mg/kg, or preoperatively at 0.3 or 1.8 mg/kg) was tested vs. once-daily enoxaparin (40 mg subcutaneous) and twice-daily apixaban (2.5 mg oral) [62] to prevent the incidence of VTE (assessed between 10 and 13 days postoperatively with bilateral venography or confirmed symptomatic deep vein thrombosis or pulmonary embolism). Postoperatively osocimab administration was noninferior at all, but the lowest dose vs. enoxaparin (VTE rates of 18%, 8%, 13%, and 14% vs. 20%, respectively). Preoperative osocimab dosing at 1.8 mg/kg was in fact superior to enoxaparin in preventing VTE (9% vs. 20% VTE, respectively), but also had the highest rate of major or clinically relevant nonmajor bleeding (4.7%) among all osocimab doses, although it was still lower than enoxaparin (5.9%). Comparisons with apixaban (12% VTE and 2% bleeding) were exploratory, and no statistical hypothesis was defined. Given the combination of efficacy and safety, the 0.6- and 1.2 mg/kg doses of osocimab appear to be most promising for future development. A phase II study in end stage renal disease patients undergoing dialysis (n = 686) was recently completed with these two doses, but findings are not yet available (NCT04523220).

3. Xisomab 3G3 (AB023).

This is a human IgG2b monoclonal antibody that binds to the apple 2 domain of FXI and FXIa and inhibits the activation of FXI by FXIIa. Despite suppressing the FXIIa-mediated activation of FXI, it leaves intact the ability of thrombin to reciprocally activate FXI, as well as the enzymatic active site of the formed FXIa itself. In a small (n = 24) study with ESRD patients on chronic hemodialysis, there were fewer occlusive events requiring hemodialysis circuit exchange and lower levels of thrombin-antithrombin complexes and C-reactive protein after xisomab administration compared with data collected prior to dosing [63]. Another phase II trial to assess the efficacy of xisomab in preventing catheter-associated thrombosis in cancer patients receiving chemotherapy is currently underway (NCT04465760).

4. Other Antibodies in Clinical Testing.

Other agents from different manufacturers are also in early stages of clinical development. These include MK-2060, which has a placebo-controlled phase II study actively recruiting to evaluate the efficacy and safety in 489 patients with ESRD on hemodialysis (NCT05027074), and REGN9933, with a phase I, placebo-controlled PK and PD study recruiting healthy participants (NCT05102136).

### 5.4. Aptamers

Aptamers are single-stranded oligonucleotides that act as potent antagonists by binding to their target protein. A number of specific aptamers have been developed that serve as strong anticoagulants by disrupting complex interactions on their target proteins [69].

To date, aptamers targeting FXI directly or indirectly are in very early stages, with none reaching clinical development. In laboratory testing, an agent designated Factor ELeven Inhibitory APtamer (FELIAP) was shown to competitively inhibit FXIa-catalyzed FIX activation and complex formation with antithrombin, without affecting FXI activation itself. Plasma clotting and thrombin generation assays were also inhibited by this aptamer [70]. Similarly, two aptamers, designated 11.16 and 12.7, that bind to sites on the FXIa catalytic domain were shown to non-competitively inhibit FXIa activation of FIX in laboratory testing [71]. In human plasma, aPTT clotting time was also significantly prolonged by aptamer 12.7.

One of the advantages of this class of agents is that it allows the possibility of developing specific antidotes that bind to its target aptamer and disrupt its aptamer–protein interaction [72]. Furthermore, a universal antidote can also be developed that blocks the action of any aptamer [73]. Despite some promising early data, the potential of aptamers seems outmatched by that of the more direct inhibitors, including monoclonal antibodies and small-molecule inhibitors. Research with these agents has thus lagged behind and even declined, but their further development as an additional therapeutic tool remains relevant.

## 6. Fields for Therapeutic Investigation

### 6.1. Active Areas of Investigations

Inhibitors of FXI/FXIa are being investigated as alternatives to standard anticoagulation therapy with heparins, VKAs, and DOACs. As such, the active areas of investigations include the usual indications for anticoagulants.

#### 6.1.1. Atrial Fibrillation

It is the most common clinically significant arrhythmia [74], with an age-related risk of occurrence, and cardiac thrombus formation and systemic embolization are its most significant clinical complications, raising the risk of stroke by 4–5 fold [75,76]. The DOACs have shown better results than warfarin in preventing stroke in non-valvular AF patients, with lower or equivalent rates of bleeding complications [77]. However, the need for safer agents still persists and is even more pressing in AF patients requiring hemodialysis. There is uncertainty as to whether the benefits of VKA actually outweigh their harm in AF patients requiring hemodialysis, and trials investigating the role of DOACs in this population are mostly in the early stages. Even in the absence of AF, hemodialysis on its own is a major problem, with cardiovascular events accounting for nearly half of the mortality in these patients. The availability of a newer antithrombotic agent with a better safety profile than existing strategies could significantly improve clinical outcomes in AF patients with or without the need for hemodialysis and in those who require dialysis, with or without AF. An FXI-inhibiting strategy could be an improved therapeutic option in these patients and warrants investigation in clinical trials.

#### 6.1.2. Venous Thromboembolism

Anticoagulant therapy is the mainstay for the prevention and treatment of VTE diseases. The development of DOACs has improved the management of VTE compared to where it was with LHMH/VKA [78]. As a result, rates of idiopathic VTE appear to be on the decline, but the incidence of non-idiopathic DVT and PE seem to be steady or increasing [79], highlighting the need for newer treatment options. Even when used at reduced doses, there is a risk of bleeding with DOAC therapy in these patients [80]. Strategies with longer-acting FXI inhibitors such as ASO and monoclonal antibodies could prove to be better treatment alternatives given their greater effect in reducing thrombosis versus impeding hemostasis.

### 6.2. Potential Areas for Therapeutic Investigations

Inhibitors of FXI/FXIa are currently in the early stages of clinical development, and over time the spectrum of their clinical application will evolve into specific, focused indications. The areas for the investigation of their therapeutic applications potentially include any pathology where thromboembolism plays an important role. Given the wide-ranging times of their onset and duration of action, FXIa inhibitors have the potential to develop into therapeutic strategies for the treatment and prevention of both acute and chronic, venous, and arterial thromboembolic disorders.

#### 6.2.1. Antiphospholipid Syndrome

Antiphospholipid antibody syndrome (APS) often manifests with symptoms of arterial and venous thrombosis, with DVT being the most common venous presentation. Current management of APS-related VTE is the same as any VTE and involves anticoagulation with heparin, followed by warfarin. Among the DOACS, rivaroxaban has been compared against warfarin to treat patients with thrombotic APS (RAPS study) but did not reach the non-inferiority threshold for the study’s primary outcome (endogenous thrombin potential—ETP) [81]. A larger trial of rivaroxaban versus warfarin was terminated early due to “unbalance in the composite endpoint between arms” without further information (TRAPS study; NCT02157272). Another trial for secondary prevention of thrombosis with apixaban in APS patients (ASTRO-APS; NCT02295475) is currently ‘Active, not recruiting’ [82]. Prevention of the thrombotic complications in APS may be a potential therapeutic area to explore using the new anti-FXI agents.

#### 6.2.2. Sickle Cell Disease (SCD)

Sickle cell disease is an autosomal recessive disorder of hemoglobin β-chain, often manifesting as chronic anemia or acute vaso-occlusive crises. Stroke is a major complication of SCD, with a prevalence rate of at least 11% in SCD patients by the age of 20 years [83]. Although the pathophysiology of stroke in SCD is not fully understood, the association is well established [84,85]. A number of variables are thought to play a role, including inflammation and TF derived from endothelial cells and monocytes, that increase the propensity for thrombosis in these patients [86,87,88,89]. Periodic red cell transfusion is the only intervention proven to prevent stroke in SCD patients in randomized trials [90]. Although it may be premature to test the benefits of FXI-inhibiting strategies in SCD patients in large-scale clinical trials, pre-clinical studies to explore treatment effect on the elevated thrombotic tendency of this population may be warranted.

#### 6.2.3. Implantable Devices/Blood Contact with Artificial Surfaces

Implantable devices that come into contact with blood, such as stents and mechanical heart valves, left ventricular assist devices, and indwelling central venous lines and ports used in chemotherapy, are frequently associated with thrombosis. Contact of blood with artificial surfaces in extracorporeal membrane oxygenation (ECMO) also causes frequent thrombotic complications. Interestingly, thromboembolism is the second major complication reported with ECMO, surpassed only by bleeding [91]. Systemic anticoagulation is recommended in ECMO, though this may be undesirable in patients at high risk of bleeding [92]. This in turn can lead to the failure of these devices and life-threatening consequences. The success rate of DOACs in preventing thrombotic events in patients with implanted devices has so far been disappointing. Not only are the DOACs non-viable treatment options in patients with devices, but are also in fact contraindicated in patients with mechanical heart valves, where warfarin is still the anticoagulant of choice. Some DOAC trials in patients with devices were terminated due to higher thrombotic and bleeding events in treated patients [93], while others were stopped due to safety reasons (NCT02872649).

Mechanical devices initiate coagulation through the contact pathway by activating FXII, leading to the local generation of TF [94]. Depletion of FXI in in vitro experiments have been shown to abolish this thrombin generation [95]. Dabigatran has been less successful in this application than warfarin in both basic and clinical testing, and given their mechanism of action, FXa inhibitors are unlikely to fare any better. None of the DOACs are approved for preventing thrombotic complications in patients with mechanical valves. FXI-directed strategies theoretically may be the most suitable for device-related treatment scenarios as they may present comparable efficacy to warfarin with a better safety profile. Prevention/treatment of thrombosis related to implantable devices appears to be one area perfectly suited for FXI-inhibiting agents and needs clinical investigation.

#### 6.2.4. Myocardial Infarction

The mainstay of CAD treatment is dual antiplatelet therapy with aspirin and one of the P2Y_12_–receptor inhibitors (clopidogrel, ticagrelor, or prasugrel). Among the DOACs, rivaroxaban is the only one to successfully undergo phase III evaluation in ACS patients in combination with dual antiplatelet therapy. It reduced the risk of death from cardiovascular causes, myocardial infarction, and stroke, but increased the risk of major bleeding and intracranial hemorrhage [96]. FXI-directed strategies could prove to be safer than rivaroxaban in ACS patients. Not only could they block contact activation on stents, but could also prevent FXI-mediated thrombus stabilization and growth.

## 7. Conclusions

Recent advances in the understanding of the contact pathway, especially of its significant role in thrombus stabilization and growth vs. in the initiation of clot formation, have opened up new targets for therapeutic intervention. FXI is one such promising target. Existing DOACs have improved treatment options compared to the classic heparins and VKA, but the bleeding risks associated with their use are substantial enough to expand the focus onto the development of their antidotes. Early indications are that FXI-directed strategies could offer similar protection against thrombotic events as DOACs, but with the added benefit of lower bleeding risk. Furthermore, the spectrum of modalities for FXI inhibition presents a range of options in both types of administration and duration of effect. With the possibility of once- or twice-monthly injections, some FXI-directed agents could also improve treatment compliance compared to current therapies. Altogether, FXIa inhibitors could be a therapeutic option in a broad spectrum of clinical scenarios that should be investigated in human trials. 

## 8. Future Directions

The broad spectrum of strategies available to modulate FXI/FXIa, including ASOs, small molecules, antibodies, and aptamers, present opportunities to explore therapeutic indications applicable in a wide variety of clinical scenarios. Several of the FXI-directed agents discussed in this review are currently undergoing clinical evaluations in phase II and phase III trials.

In addition to investigating the effectiveness of FXI-directed strategies versus anticoagulants (i.e., heparins, warfarin, and DOACs), their safety and efficacy should also be assessed in combination with anti-platelet agents because a large swath of the population is on chronic aspirin therapy with or without a P2Y_12_-receptor inhibitor.
